# Comprehensive Look at Blood Transfusion Utilization in Total Joint Arthroplasty at a Single Academic Medical Center under a Single Surgeon

**DOI:** 10.1155/2013/983250

**Published:** 2013-07-16

**Authors:** Sean Robinson, Owen McGonigle, Sam Volin, Yung-Chi Sun, Matthew Moore, Charles Cassidy, Eric Smith

**Affiliations:** Department of Orthopaedic Surgery, Tufts Medical Center, Boston, MA 02111, USA

## Abstract

The utilization of autologous and allogeneic transfusions in total joint arthroplasties was to characterize patients who may benefit from giving preoperative blood donations. We conducted a retrospective chart review of 525 patients to document preoperative hematocrit, estimated blood loss, length of stay, transfusions, and medical comorbidities. Results of our review showed that total hip arthroplasty revision (THA-R) had the highest prevalence of transfusions (60%) followed by total hip arthroplasty (THA, 53%), total knee arthroplasty-revision (TKA-R, 33%), and total knee arthroplasty (TKA, 23%). There was significant waste of autologous donations: 92% of TKA patients, 64% of THA, and 33% of THA-R patients wasted on average 1.527, 1.321, and 1.5 autologous units, respectively. Pre-operative hematocrit was the strongest predictor of future transfusion need across all procedures, and primary THA had additional predictors in age and gender.

## 1. Introduction

Anemia is a significant and frequent complication of total joint replacements. Anemia after total joint replacement has been shown to increase length of stay, decrease immediate postoperative physical function, and increase the likelihood of requiring a blood transfusion [1]. Allogeneic blood transfusion, while important in treating anemia, are not without risks and have been shown to lead to immunosuppression, blood-borne disease transmission, immunologic reactions, allergic reactions, and increased mortality [1–3].

Developing strategies to treat or prevent postoperative anemia while limiting the exposure of patients to allogeneic blood has become an important focus. Multiple studies have found that low preoperative hemoglobin is a major risk factor for perioperative and postoperative transfusion after total joint arthroplasty [4–7]. Attempts to improve preoperative hematocrit, including iron therapy and erythropoietin stimulating agents, have been inconsistent in demonstrating a significant effect on preoperative hematocrit [8]. Additional strategies have included perioperative blood salvage (i.e., cell saver), hemodilution, and preoperative donation of blood for autologous transfusion [9–12].

Using autologous donations for transfusion avoids many of the adverse events associated with allogeneic transfusion as noted above [2, 13]. Autologous donations, if utilized effectively, decrease the cost of obtaining, storing, and using allogeneic blood [14–16]. In practice, however, obtaining preoperative autologous donations is often time consuming, expensive, and inefficient. Additionally, many patients who donate will not require a transfusion [4, 9, 11]. In their study of 9,482 patients, Bierbaum et al. concluded that the utilization of autologous blood is inefficient and the process of determining those who may need transfusion is underdeveloped [4]. Understanding which patients are most at risk of developing anemia after total joint replacement is important for optimal preoperative, intra-operative, and postoperative care, as well as maximal utilization of blood resources.

The purpose of this study was to review the blood transfusion utilization of patients undergoing total knee arthroplasty (TKA), total knee arthroplasty revision (TKA-R), total hip arthroplasty (THA), and total hip arthroplasty revision (THA-R) at a single academic institution under a single surgeon. We evaluated this cohort for potential risk factors that may predispose one to a transfusion and documented the usage and waste of preoperative autologous blood donations. To our knowledge, no studies have evaluated data from a single surgeon and institution with a uniform protocol from preoperative evaluation through postoperative followup. This study may provide a unique perspective that eliminates confounding factors that may arise in studies that include multiple surgeons with many different protocols. 

## 2. Materials and Methods

A retrospective chart review was conducted of all TKA, TKA-R, THA, and THA-R preformed from March 2009 to March 2011 at a single academic medical center with the senior author performing all procedures. Approval was granted from the Institutional Review Board. In total, 526 patients were enrolled in the study: 246 total knee arthroplasties, 155 total hip arthroplasties, 67 total knee revisions, and 58 total hip revisions. 

The senior author's protocol for patients in this study started with a preoperative clinic visit where the decision was made to proceed with surgery. Preoperative evaluation for all patients included an anesthesia consult, EKG, chest X-ray, and standard preoperative labs including a complete blood count, chemistry profile, type and screen, prothrombin time and INR. Additional labs or studies were ordered on selected patients if deemed appropriate based on history or physical exam. 

Autologous donations were collected two weeks prior to the operation for patients who were thought to be at increased risk for blood transfusions. Determination of risk was individualized and included factors such as preoperative hematocrit, medical comorbidities, age, gender, general health, and patient's desire to donate. See Table 3 for details regarding the average number of units of autologous packed red blood cells donated for each procedure. Each unit collected was 220 cc. 

On the day of surgery, all primary and revision total knee arthroplasties had a thigh tourniquet placed and inflated (tourniquet pressures varied from 275 to 325 mmHg based on thigh size) prior to skin incision. At the time of closure, a hemovac drain was placed in all primary and revision total knee arthroplasties. There were no drains used in any primary or revision total hip arthroplasties. Upon admission to the post anesthesia care unit (PACU), all patients had a hematocrit drawn. Patients were transferred to the surgical ward as per PACU protocol. Hematocrit (HCT) values were measured daily during the patient's hospital stay. Hemovac drains were removed on postoperative day 1 regardless of drain output. Patients were discharged to home or rehab based on evaluation by the arthroplasty service in coordination with physical and occupational therapy. The decision to transfuse during the hospital stay was based on a global evaluation by the arthroplasty service which included patient symptoms, hematocrit value, vital signs, urine output, and estimated blood loss in surgery. There was no cut-off hematocrit below which a transfusion was required, or value above which no transfusion could be given; however, a hematocrit of 25.5% or less was used as a cut-off guide for most patients. 

Data for evaluation in this study was obtained by reviewing clinical charts, anesthesia records, and patients' hospital records. Information recorded from the clinical chart included the procedure, preoperative hematocrit value and patient co-morbidities. Comorbidites were grouped into general categories which included cardiovascular, gastrointestinal, hematology/oncology, pulmonary, renal, musculoskeletal, endocrine—other than diabetes mellitus (DM), diabetes mellitus, psychiatric illnesses, neurologic disease (including seizures, history of stroke), liver disease, gynecologic disorders, dyslipidemias, and autoimmune disorders. The patients hospital record and anesthesia record were evaluated for hematocrit values, hematocrit value at which the patient was transfused, number of units transfused, number of allogeneic transfusions, number of autologous transfusions, number of wasted autologous units, and additional breakthrough transfused units beyond the autologous donation. Complications occurring both intraoperatively and postoperatively were also recorded. 

### 2.1. Statistical Methods/Analysis of Data

 General demographics were first determined with an average and standard deviations (Table 1). The total transfusion data was determined and further stratified by allogeneic transfusions, autologous transfusions, and breakthrough transfusion requirement (Tables 2 and 3). Two comparative groups were created: transfused patients versus nontransfused patients (Table 4) and patients who gave an autologous donation versus patients who did not (Table 5). Age, length of stay (LOS), preoperative HCT, estimated blood loss (EBL), 14 comorbidities were compared, and a *P* value was assigned to each comparative group. Logistic regressions were conducted to predict patient transfusion using age, gender, and pre-op HCT as predictors. A test of the full model against a constant only model was conducted for each of the four groups (THA, TKA, THA-R, and TKA-R) to determine the predictors of transfusion. Additionally, coefficient of determination (*R* ^2^), Wald's criteria, and EXP(*B*) were used to determine the chance of future events (transfusion) for each variable: preoperative hematocrit, age, gender, and comorbidities.

## 3. Results

### 3.1. TKA

A logistic regression analysis was conducted to predict patient transfusion for 246 patients. A test of the full model against a constant only model indicated that preoperative hematocrit was a significant predictor of transfusion (chi square = 27.866, *P* < 0.0001 with df = 1). Age, gender, and comorbidities were not found to significantly predict transfusion (*P* > 0.05). 

Nagelkerke's *R* ^2^ of 0.173 indicated a small positive relationship between Preoperative hematocrit and transfusion. Prediction success overall was 81.1%. The Wald criterion demonstrated that preoperative HCT made significant contributions to prediction (*P* < 0.0001). EXP(*B*) value indicates that when preoperative HCT is decreased by 1% the odds ratio is 1.258, and therefore patients are 1.258 more likely to need transfusion.

### 3.2. TKA-R

A logistic regression analysis was conducted to predict transfusion for 67 patients. A test of the full model against a constant only model indicated that preoperative hematocrit was a significant predictor of transfusion (chi square = 11.264, *P* < 0.005 with df = 1). Age, gender, and comorbidities were not found to significantly predict transfusion (*P* > 0.05). 

Nagelkerke's *R* ^2^ of 0.216 indicated a small positive relationship between preoperative HCT and transfusion. Prediction success overall was 70.1%. The Wald criterion demonstrated that pre-op HCT made significant contributions to prediction (*P* < 0.005). EXP(*B*) value indicates that when pre-op HCT is decreased by 1%, the odds ratio is 1.203, and therefore patients are 1.203, more likely to need transfusion.

### 3.3. THA

A logistic regression analysis was conducted to predict patient transfusion for 155 patients. A test of the full model against a constant-only model indicated that age, gender, and preoperative hematocrit significantly predicted transfusion (chi square = 35.141, *P* < 0.0001 with df = 3). Comorbidities were not found to significantly predict transfusion (*P* > 0.05). 

Nagelkerke's *R* ^2^ of 0.279 indicated a small positive relationship between the predictors (age, gender, and preoperative HCT) and prediction (transfusion or not). Prediction success overall was 70.7%. The Wald criterion demonstrated that age, gender, and preoperative HCT made significant contributions to prediction (*P* < 0.05). EXP(*B*) value indicates when age is raised by one unit (one year), the odds ratio is 1.029, and therefore patients are 1.029 more times likely to need transfusion. In addition, EXP(*B*) value indicates that for female patients the odds ratio is 2.326, and therefore female patients are 2.326 more times likely to need transfusion. Finally, EXP(*B*) value indicates that when preoperative HCT is decreased by 1%, the odds ratio is 1.236 and therefore patients are 1.236, more likely to need transfusion.

### 3.4. THA-R

A logistic regression analysis was conducted to predict patient transfusion for 58 patients. A test of the full model against a constant only model indicated that preoperative hematocrit significantly predicted transfusion (chi square = 5.896, *P* < 0.05 with df = 1). Age, gender, and comorbidities were not found to significantly predict transfusion (*P* > 0.05). 

Nagelkerke's *R* ^2^ of 0.133 indicated a small positive relationship between the preoperative HCT and transfusion. Prediction success overall was 64.9%. The Wald criterion demonstrated that preoperative HCT made a significant contribution to prediction (*P* < 0.05). EXP(*B*) value indicates that when preoperative HCT is decreased by 1%, the odds ratio is 1.206, and therefore patients are 1.206 more likely to need transfusion.

## 4. Discussion

 This study reviewed the utilization of blood transfusion after total joint arthroplasty at a single academic medical center performed by a single orthopaedic surgeon. Data collected was analyzed for preoperative risk factors that are predictive of transfusion as well as how preoperative autologous blood donations were utilized. In a previous study, Bierbaum et al. analyzed similar blood usage but among a cohort of 330 orthopaedic surgeons spread over various geographic locations throughout the United States. They determined that preoperative hemoglobin and lack of pre-donated units were the most consistent predictors of allogeneic transfusion need [4]. They also concluded that autologous blood donation and utilization was grossly inefficient. Bierbaum et al. noted a limitation in the lack of a uniformed protocol among surgeons participating whereas the present study has the advantage of a consistent protocol from preoperative visit to postoperative followup.

Logistic regression analysis of our collected data demonstrated preoperative hematocrit as a consistent predictor of future transfusion—similar to several other studies [4, 9, 11, 12]. For each one-point decrease in preoperative hematocrit, the odd ratio demonstrated an increased possibility for transfusion for all procedures (OR 1.203–1.258). These odds ratios are additive, and, therefore, those patients with a hematocrit several points lower than normal had a much higher risk of transfusion. In addition to preoperative hematocrit, predictors of transfusion for total hip arthroplasty (THA) included age and gender. For each year increase in age, the chance of transfusion increased by 1.029. Additionally, female patients were more likely to receive transfusion with an odds ratio of 2.326. This relationship, to our knowledge, has not been demonstrated in previous studies. 

Preoperative hematocrit was equally shown to be a predictor of future transfusion in total knee arthroplasty (TKA), total knee arthroplasty revision (TKA-R), and total hip arthroplasty-revision (THA-R) groups; however, no other variables (age, gender, and comorbidities) were found to be significant predictors for transfusion. It is unclear why age and gender were found to be significant only in THA patients. 

A review of our overall blood usage (both allogeneic and autologous) shows that we were frequently unsuccessful in predicting those who will require a transfusion. In all, THA-R had the greatest proportion of patients receiving transfusions followed closely by THA, TKA-R, and TKA. While TKA had the fewest patient requiring transfusion in all, this group had 62 patients (25%) providing an autologous donation. In addition, only 13 (21%) of those patients providing donation actually required transfusion; therefore, the vast majority of donators wasted their entire donation. This waste accounts for a large fee in blood draw, storage, and retrieval [14–16]. On the other hand, zero patients provided a donation in the TKA-R arm of the study, but 33% of patients required allogeneic blood (average of 2.7 units). As for THA and THA-R, these groups had the highest percentage of patients requiring a transfusion (53% and 60%, resp.) and required the most units of blood (154 units and 124 units, resp.), but only 29% and 11% provided an autologous donation, respectively. Inconsistency in choosing appropriate patients for pre-donation is not limited to our study; similar difficulty has been documented in other studies [4, 9, 12]. 

Ultimately better models are needed to predict the patients who are most and least likely at risk for needing a transfusion in the perioperative period. Improved patient selection will help reduce risks to the patient, decrease wasted blood, and lower expenses. 

Shortcomings of this study include its nonrandomized nature. We did not perform a power analysis to determine the number of charts to review, so it is unclear if collecting more data might allow trends we found to become significant. While we attempted to extract information from our chart review believed to be important preoperative risk factors, additional information could always be considered for analysis. Future reviews could include BMI, race, or history of previous transfusion requirements. In addition, comorbidities were grouped into broad categories rather than evaluating specific conditions. This may have hidden the role that certain disease processes play in predisposing patients to anemia after surgery, particularly in less commonly seen diseases. Future evaluation looking at the effect of specific diseases would be beneficial. To do this, however, we would likely need a much larger patient database for proper power.

Finally, there was no specific protocol for transfusion of patients. While the same generalized principles were applied to all the patients in the decision to transfuse, results may have been affected because there was no uniform outline for when patient should or should not be transfused. While theoretically possible to implement, this may be difficult based on the number of factors to consider when deciding to transfuse. 

Despite these flaws, we have provided the first comprehensive summary of blood transfusion utilization at a single academic institution under a single surgeon. Our results confirmed previous findings that that preoperative hematocrit is the most significant factor that determines need for postoperative blood transfusion. In addition, our data suggests that age and gender, particularly in THA, may play an important role. The inefficiencies of autologous blood collection and utilization were again demonstrated. Continued analysis of additional variables (BMI, race, and more specific comorbidity data with a scoring system such as the Charlson comorbidity score [17]) may better stratify and identify those patients most at risk for transfusion.

## Figures and Tables

**Table 1:** General demographics.

			Gender		Age			
					Mean (range)			Range
			Men	Women	Total	Men	Women	
TKA	Total patients	246	92	154	62.7	61.5	63.5	21–88
	*Unilateral *	241						
	*Bilateral *	5						
TKA-R	Total patients	67	22	45	63.8	69.7	61	30–85
	*Unilateral *	65						
	*Bilateral *	2						
THA	Total patients	155	70	85	59.1	56.6	61.3	19–86
	*Unilateral *	152						
	*Bilateral *	3						
THA-R	Total patients	58	33	25	56.7	55.3	58.5	26–91
	*Unilateral *	57						
	*Bilateral *	1						

	Total	526	217	309	61.1	59.8	62.1	19–91

**Table 2:** General transfusion data.

			Number of patients (%) requiring transfusion			Total units			Units transfused
	Pre-op HCT	HCT on day of transfusion	Total	Women	Men	Total	Allogeneic	Autologous	Avg. units per patient
TKA	35.8	24.8	54 (22%)	41	13	79	62	17	1.4
TKA-R	37.2	24.9	22 (33%)	12	10	60	60	0	2.7
THA	38.8	24.0	82 (53%)	56	26	154	111	43	1.9
THA-R	38	24.5	35 (60%)	15	20	124	119	5	3.5

**Table 3:** Autologous blood donation and utilization.

	Total donators (%)	Units donated	Donators who required transfusion	Patients who wasted total donation	Avg. donated units wasted	Patients with breakthrough transfusions	Avg. breakthrough units
TKA	62 (25%)	1.65	13	80%	1.3	7%	2
TKA-R	0	0	0	0%	0	0	0
THA	45 (29%)	1.5	22	51%	1.4	32%	1.6
THA-R	6 (11%)	1.3	4	33%	1.5	0	0

**Table 4:** Transfused versus Nontransfused patients.

		TKA	*P* value	TKA-R	*P* value	THA	*P* value	THA-R	*P* value
Age	*Not transfused *	62.0	0.007	61.3	0.004	56.1	0.07	52.8	0.06
	*Transfused *	65.1		69.0		61.8		59.2	
LOS	*Not transfused *	3.4	0.03	3.8	0.09	3.5	0.04	4.0	0.43
	*Transfused *	3.8		4.6		4.1		5.7	
Pre-op HCT	*Not transfused *	40.5	0.001	38.7	0.001	40.4	0.001	40.5	0.008
	*Transfused *	37.3		34.3		37.4		36.5	
EBL	*Not transfused *	57	0.05	96	0.008	279	0.03	736	0.003
	*Transfused *	75		285		349		1326	
Comorbidities	*Not transfused *	2.92	0.2	2.08	0.07	2.52	0.07	2.29	0.002
	*Transfused *	3.07		3.27		2.74		3.03	

**Table 5:** Autologous donation patient versus Nondonation patients.

		TKA	*P* value	THA	*P* value	THA-R	*P* value
Age	*Auto donation *	57.4	0.001	54.1	0.001	49	0.1
	*No auto donation *	64.5		61.1		57.6	
Pre-op HCT	*Auto donation *	39.0	0.01	37.8	0.03	39.7	0.2
	*No auto donation *	40.1		39.2		37.9	
EBL (mL)	*Auto donation *	64.2	0.2	331.3	0.3	845	0.2
	*No auto donation *	59.8		310.0		1120.4	
Comorbidities	*Auto donation *	2.7	0.1	2.4	0.09	3.3	0.2
	*No auto donation *	3.0		2.7		2.8	
